# Mitochondrial genome-derived microsatellites reveal genetic diversity and population structure in Callery pear populations

**DOI:** 10.1371/journal.pone.0358231

**Published:** 2026-09-17

**Authors:** Alina Pokhrel, Denita Hadziabdic, Robert N. Trigiano, William E. Klingeman, David R. Coyle, Marcin Nowicki

**Affiliations:** 1 Department of Entomology and Plant Pathology, University of Tennessee, Knoxville, Tennessee, United States of America; 2 Department of Plant Sciences, University of Tennessee, Knoxville, Tennessee, United States of America; 3 Department of Forestry and Environmental Conservation, Clemson University, Clemson, South Carolina, United States of America; Anhui University of Chinese Medicine, CHINA

## Abstract

Callery pear (*Pyrus calleryana* Decne.; PC) possesses many desirable characteristics valued in managed landscapes. This has driven the release of numerous cultivars, including both hybrids and selections derived from native populations. The extensive planting of PC cultivars in managed areas has contributed to the widespread occurrence of invasive individuals across a broad range of habitats in the eastern United States (US). Self-incompatibility, tolerance to various environmental conditions, pathogen and pest resistance, intraspecific hybridization among the cultivars, possible interspecific hybridization with other *Pyrus* species, and seed dispersal by various vertebrates have contributed to the spread and persistence of PC across diverse environments. Because effective and environmentally appropriate management options remain limited, improved understanding of PC genetics may help inform management strategies. Previous studies have characterized PC diversity using nuclear genomic short sequence repeats (gSSRs), however, neither a mitochondrial genome resource nor mitochondrial short sequence repeats (mtSSRs) have been developed for this purpose. Here, we assembled a mitochondrial genome of 485,892 bp and used five mtSSRs to characterize mitochondrial diversity and population structure among accessions from the species’ native range in Asia (*n* = 72), southeastern US escapees (SNesc; *n* = 90), Tennessee escapees (TNesc; *n* = 90), and US-released commercial cultivars (UScult; *n* = 69 representing 14 unique cultivars). We found a high genetic diversity (H_e_ = 0.728) and evidence of genetic structure in PC. In distance-based and multivariate analyses, UScult occupied an intermediate position between the Asian populations and the US escapees. The observed mitochondrial diversity among samples assigned to PC cultivars is consistent with a complex genetic landscape and may reflect distinct maternal lineages, cultivar-labeling or record-keeping discrepancies, and/or technical variation. This study underscores the need for broader genomic investigations using authenticated cultivar reference material and high-resolution nuclear markers to resolve cultivar ancestry, validate true-to-name identity, and inform species management.

## Introduction

Callery pear (*Pyrus calleryana* Decne. [PC]) is a flowering pear tree native to Asia that was introduced to the United States (US) between 1908 and 1919 for use as a breeding resource. The introduction was made with the intention of protecting the edible European pear (*P. communis* L.) from fire blight, a bacterial disease caused by *Erwinia amylovora* (Burril) [1,2]. In 1952, USDA breeder John Creech recognized the ornamental potential of PC after observing a 33-year-old tree with a globular crown, glossy foliage, abundant flowers, and few spurs [3,4]. Using this tree as a scion source for grafting onto another PC rootstock, the first ornamental cultivar, ‘Bradford’, was released to the commercial market in 1962, quickly becoming one of the most widely planted street trees in the eastern US [1,5]. The widespread popularity of the Bradford pear prompted the development and release of at least 25 additional ornamental cultivars that were derived from various sources of PC accessions, including Asian seed collections and several other, often unknown or undocumented parents [6,7].

PC is self-incompatible and rarely sets viable fruit in its native range; thus, its cultivars were initially considered to have limited invasive potential [1,4,8,9]. But, when multiple cultivars of PC or other *Pyrus* species are planted together or occur nearby, cross-pollination among genetically diverse material can produce abundant, viable seed [6,7,10]. After the fruit is softened by frost, birds and wildlife readily consume it and disperse the seeds [4,11]. Escaped PC populations, first noted in 1964, now occur in at least 40 US states [12], and encroach forest edges, roadsides, grasslands, creek and streambeds, riverbanks, and other disturbed early successional habitats [1,7,13]. Collectively, the release of new PC cultivars and the practice of grafting provided the trees with sufficient genetic variation to facilitate cross-pollination and form self-sustaining populations [14–16]. In addition, traits such as high seed viability for up to 11 years, high germination rates of 45–87% [17,18], early reproductive maturity, prolific flowering and fruiting [4,8], resistance to pathogens, pests, and herbivores [19], extended leaf phenology, and broad environmental tolerance contributed to invasion of PC into a variety of environments [20–22]. High genetic diversity and gene flow may increase the evolutionary potential of introduced PC populations in new environments [13].

PC is now considered a noxious weed in many eastern US states, and few effective management strategies exist. The most effective management of PC requires the use of herbicides, though the specific application technique depends on the size of the tree [9,23–25]. In border areas and lands where prescribed fire may be feasible, fire has been ineffective as a stand-alone PC management technique [25,26] yet can help reduce the likelihood of damage from PC thorns [14]. Management challenges occur because PC will vigorously resprout after either freezing or fire [20,27], thus necessitating the use of herbicides for effective control. Because of PC’s resilience in the landscape, a deeper understanding of the genetics of this invasive species is essential for improving management strategies, as genetic and evolutionary mechanisms strongly influence PC establishment and spread [28].

The genomic basis of adaptation and invasiveness of introduced plant species has remained largely unexplored, primarily due to the limited availability of genomic resources [29]. Few studies have evaluated PC’s genetic diversity in its native range [11,30,31], and relatively few studies have examined escaped populations in the US [13,15,32]. Microsatellite markers or short sequence repeats (SSRs) have been used in population genetics and phylogenetics studies of different plant species [33–35]. SSRs are characterized by high repetitiveness and mutation rates, extensive polymorphism and reproducibility, and provide useful information for analyzing genetic diversity, genetic relationships, and population structures of plants [36–38]. The typically maternal inheritance, haploid state, and limited recombination of mitochondrial DNA (mtDNA) as compared to nuclear DNA (nrDNA) make mitochondrial short sequence repeats (mtSSRs) useful for assessing maternal-lineage diversity and phylogeographic structure [38]. Previous investigations have utilized nuclear genomic short sequence repeats (gSSRs) to assess the genetic diversity of PC, however, neither the mtDNA nor the mtSSRs of PC have been developed previously for use in genetic diversity assessment. Developing the mtDNA of PC would provide a valuable addition to the existing genomic resources for this species. Even though PC has been cultivated in the US for more than a century, few studies have assessed the genetic diversity and invasion potential of US commercial PC cultivars [8,11,39]. Comparison of native-range and introduced-range PC populations using mtSSRs can help describe differences in maternal-lineage composition, although organellar markers alone cannot resolve the demographic and selective processes underlying invasion [40]. Population-genetic analyses can help characterize patterns relevant to invasion history and management [41]. Comparison with published gSSR results can identify concordance or discordance between biparentally inherited nuclear patterns and maternally inherited mitochondrial patterns; mtSSR data alone cannot quantify nuclear gene flow or recombination [13].

In previous work, results suggested that genetic differentiation exists between native and escaped populations [11] and that a high genetic diversity can be observed within and between PC populations across the native, introduced cultivated, and escaped naturalizing populations of PC. To further characterize differentiation among these groups, we undertook this research with the objective of characterizing the genetic diversity of Asian populations, naturalized US-escapes, and US commercial cultivars of PC by using mtSSR-based genotype data. Characterizing these patterns may help inform and refine management strategies for PC in the eastern US.

## Materials and methods

### Sample collection

Leaf samples of *Malus rockii* Rehder and various *Pyrus* species, including Asian specimens of PC and the original US cultivar selections of PC used by [11], were used in this study. For Asian accessions lacking country-of-origin metadata (NA), we assigned a provisional most likely country of origin using a k-nearest neighbors approach based on our five mtSSR loci. Genotypes were formatted as a numeric allele matrix, with missing alleles coded as 0, and Euclidean distances were computed between samples using only shared, non-missing loci. For each NA-country sample, the five closest non-NA individuals were identified, and country of origin was assigned by majority vote (k = 5). The specimen collection was supplemented by samples from four different PC cultivars: ‘Bradford’, ‘Autumn Blaze’, ‘Aristocrat’, and ‘Cleveland Select’, which were purchased from Ty Ty Plant Nursery (Ty Ty, GA) and maintained in the University of Tennessee, Knoxville (UTK) nursery facility (35°56’46.8"N 83°56’17.6"W), and leaf samples of confirmed PC cultivars (‘Aristocrat’ ID: 475-72A, ‘Bradford’ ID: 644-65B) from Arnold Arboretum, Boston, MA (42°17′52″N 71°7′22″W 42.29778°N 71.12278°W). In total, 72 specimens were from the Asian origin, 69 were from US cultivar selections representing 14 unique cultivars (UScult) (Supplementary File 1; S1 Table). Additional documented accessions of PC and other relevant species were requested from Germplasm Resources Information Network (GRIN USDA), herbaria, and arboreta. Altogether, 89 specimens of 16 *Pyrus* species and two specimens of *M. rockii* were also used to evaluate the cross-amplification potential among the developed mtSSRs (Supplementary File 1; S2 Table). Leaf samples from naturalized escaped PC trees collected from Tennessee, Georgia, and South Carolina [13] were also used. No specific permits were required for sampling in these public locations. The samples were grouped into “North Group” (TNesc) and “South Group” (SNesc) based on the sites of sample collection. The escaped-population dataset comprised 180 samples: 90 from Tennessee (TNesc) and 90 from Georgia/South Carolina (SNesc) (Supplementary File 1; S1 Table).

### DNA extraction

Genomic DNA of the collected specimens was extracted using approximately 100 mg of dried leaf per sample. Samples were homogenized using a Bead Mill 24 (Fisher Scientific, Pittsburgh, Pennsylvania, USA) and subsequently used for DNA extraction using EZNA DNA DS Mini Kit (Omega Bio-Tek, Norcross, GA, USA), following the manufacturer’s protocol. The concentration and purity of extracted DNA were assessed using Nanodrop Spectrophotometer (Thermo Fisher Scientific, Wilmington, Delaware, USA).

### Development of the mitochondrial genome and its phylogenetic analysis

A draft mtDNA genome of PC was assembled using NOVOwrap version 3.7.2 (Wu et al., 2021). Available whole-genome paired-end Illumina sequencing data of PC accession collected in China (Jinshan Fruit Tree Test Station in Shanghai Academy of Agricultural Sciences; GenBank SRR16505594) was used for reference-guided assembly of the mitochondrial genome. The mitochondrial genome of *P. communis* (GenBank NC_065229.1) was used as a reference to guide the assembly of SRR16505594. NOVOwrap assembly yielded 16 candidate genome architectures, however, only six were provisionally consistent with a complete circular representation and were selected for further evaluation; these were further evaluated bioinformatically and confirmed using PCR and Sanger sequencing. To the latter goal, the six genome assemblies were aligned in mVISTA [42], with the longest available mtDNA of *Pyrus, P. betulifolia* mtDNA (NCBI: MW080658) used as the baseline reference genome. The alignment allowed for the identification of gaps or discrepancies present among the different putative mtDNA PC assemblies compared to that reference. The observed discrepancies among candidate assemblies indicated regions requiring experimental validation. Primers spanning unresolved or discordant assembly regions were designed using PrimerQuest (Integrated DNA Technologies (IDT), Morrisville, NC, USA), based on the identified gaps. Then, the long-range PCR conditions were optimized using touch-down and gradient PCR and final PCR reactions were performed using the optimized conditions. For smaller expected products, with sizes up to 3 kb, PCR was done in a 10 µl reaction mixture consisting of 5 µl of AccuStart II PCR supermix (2×) (Quantabio, Beverly, MA, USA), 1 µl each of 10 µM forward and reverse primers, 2 µl of sterile water, and 1 µl of 5 ng/µl DNA. For expected PCR products of > 3 kb, Platinum SuperFI PCR mastermix (Thermo Fisher Scientific, Waltham, MA, USA) was used instead of AccuStart II PCR supermix, otherwise the reaction composition remained the same. The 3 kb PCR program used for temperature optimization was: initial denaturation at 94 ℃ for 2 min, followed by 10 cycles of denaturation at 94 ℃ for 15 s, gradient annealing at 53℃ - 60 ℃ with a touch-down of 0.7 ℃/cycle for 30 sec and an extension at 72 ℃ for 3 min 30 s, followed again by 30 cycles of denaturation at 94 ℃ for 15 s, gradient annealing at 48–55 ℃ for 30 s, and an extension at 72 ℃ for 3 min 30 s, with final extension at 72 ℃ for 1 min. For primer pairs with different product sizes, the extension time was adjusted accordingly, whereas all other cycling parameters remained unchanged. The amplified PCR products were electrophoresed in 2% w/v agarose gel stained with ethidium bromide, with a 100 bp Invitrogen ladder (Thermo Fisher Scientific, Waltham, MA, USA) for smaller PCR products and Lambda DNA-HindIII Digest (New England Biolabs, Ipswich, MA, USA) as a reference for larger PCR products. The optimized thermal profile for < 3 kb products was: initial denaturation at 94 ℃ for 2 min., followed by 10 cycles of denaturation at 94 ℃ for 15 s, annealing at 56.4 ℃ for 30 s and an extension at 72 ℃ for 3 min. 30 s, followed again by 30 cycles of denaturation at 94 ℃ for 15 s, annealing at 51.5 ℃ for 30 s, and an extension at 72 ℃ for 3 min. 30 s, with final extension at 72 ℃ for 1 min. The resulting alignment was evaluated to select the best-fitting nucleotide-substitution model (GTR + G), and the phylogenetic tree was visualized in SeaView v5.0.5. The selected genome underwent further validation through Sanger sequencing of the amplified PCR products < 3 kb and the mtDNA F3B3 sequence data [43]. The final draft assembly of mtDNA was then polished using the PILON v.1.23 [44] and the SRR16505594 fastq data. Finally, annotation was done using GeSeq v.2.03 from the Chlorobox suite [45].

**Fig 1:**
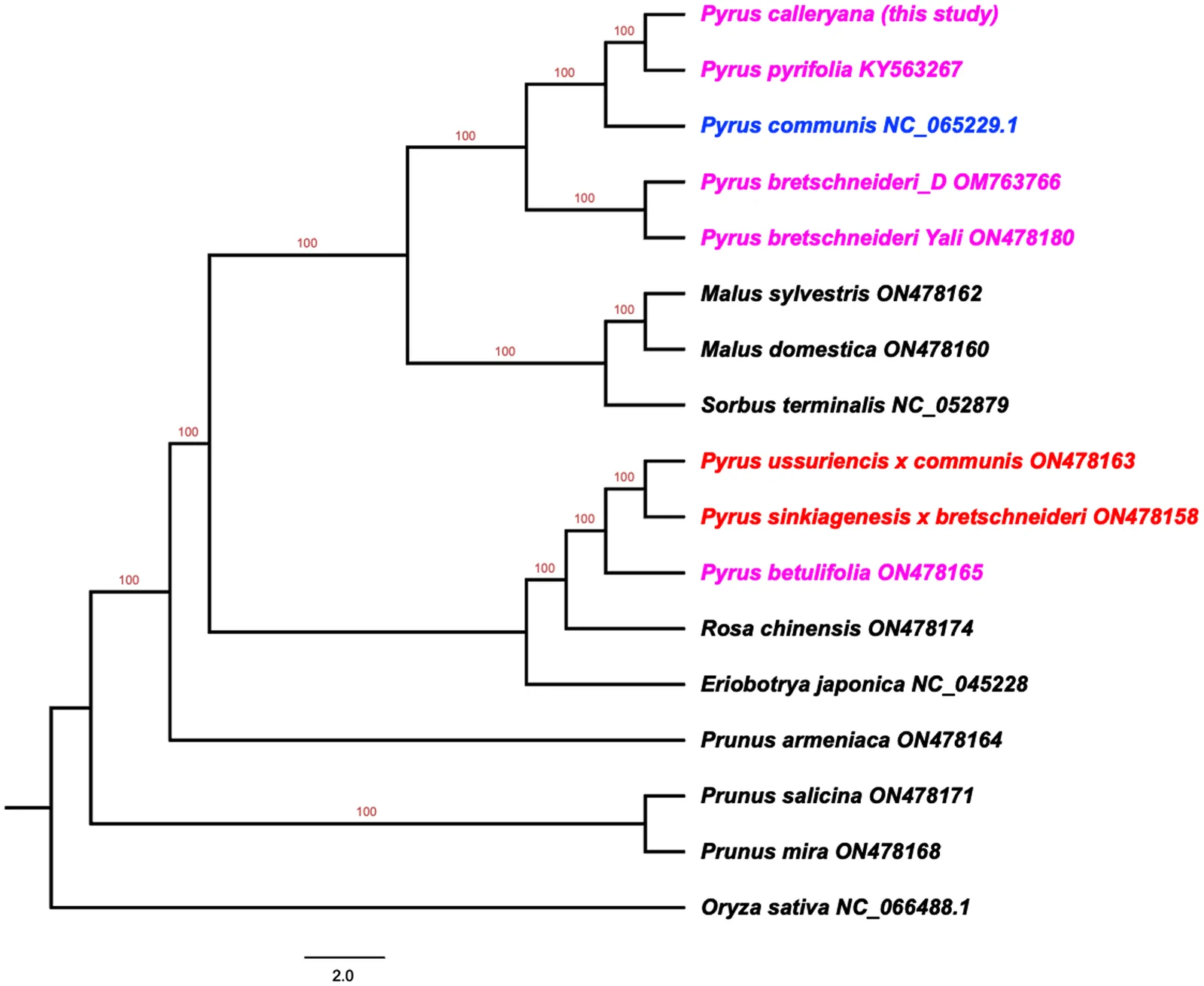
Phylogenetic placement of the newly developed mitochondrial genome of *Pyrus calleryana.* All mitochondrial genomes used for analysis are labeled with their respective GenBank IDs. MAFFT-aligned genomes were analyzed under the GTR + G nucleotide-substitution model with 100 bootstrap replicates. The tree was re-rooted using the single monocot species, *Oryza sativa*. For reference, the genetic distance legend is placed at the bottom. Colored nameplates denote the geographic origins of given *Pyrus* species: East Asia — pink; Europe — blue; Pyrus hybrids — dark red.

The phylogenetic analysis of PC complete mtDNA was based on the alignment with available mtDNA of other *Pyrus* spp., related Rosaceae spp., and the monocot *Oryza sativa* L. to root the tree. The mtDNA sequences identified by their respective GenBank accession numbers (Fig 1) were aligned using the default parameters in MAFFT v7 [46]. The resultant sequence matrix was analyzed for the best substitution model (GTR  +  G), and the tree was visualized in SeaView v5.0.5 [47].

### Mitochondrial Microsatellite markers

The final mitochondrial genome draft was screened for perfect short sequence repeats (SSRs) using USDA SSR finder v1.00 [48]. A genome-wide screen targeting perfect-repeat mitochondrial SSRs with a minimum of five repeats and motif lengths of 2–4 bp was conducted. Out of the identified SSR candidates, five mtSSR loci (67k, 150k, 333k, 353k, and 417k) were retained after screening for polymorphism, amplification performance, and primer-dimer formation. The 19- to-28-bp long SSR primers were designed using the PrimerQuest tool of IDT with 35–50% GC content, 53–55 °C melting temperature, and 100–500 bp of expected product sizes, with the SSR motif positioned approximately centrally within each expected amplicon. PCR was done in a 10 µl reaction mixture consisting of 5 µl of 2 × AccuStart II PCR supermix, 1 µl each of 10 µM forward and reverse primers, 2 µl of sterile water, and 1 µl of 2ng/µl DNA. The DNA extracted from PC sample PC_A_054 (Arnold Arboretum, accession: 156099) was used as a positive control, and sterile distilled water was used as a negative control. The gradient and touch-down PCR program used for temperature optimization of mtSSR primers was: initial denaturation at 94 ℃ for 2 min., followed by 10 cycles of denaturation at 94 ℃ for 15 s, gradient annealing at 50–60 ℃ with a touch-down of 0.5 ℃/cycle for 30 sec and an extension at 72 ℃ for 30 s, followed again by 30 cycles of denaturation at 94 ℃ for 15 s, gradient annealing at 45 ℃ - 55 ℃ for 30 s, and an extension at 72 ℃ for 30 s, with a final extension at 72 ℃ for 1 min. The amplified PCR products were subjected to agarose gel electrophoresis in a 2% w/v agarose gel stained with ethidium bromide and a 100 bp Invitrogen ladder (Thermo Fisher Scientific) as a reference. The visualization was done under UV light using UVP GelStudio PLUS (Analytik Jena, Upland, CA, USA) and documented using VisionWorks v8.22.18309.10577. The optimized thermal profile for final PCR applied across all SSR loci was: initial denaturation at 94 ℃ for 2 min., followed by 10 cycles of denaturation at 94 ℃ for 15 s, annealing at 59 ℃ for 30 s with a touch-down of 0.9 ℃/cycle and an extension at 72 ℃ for 30 s, followed again by 30 cycles of denaturation at 94 ℃ for 15 s, annealing at 50 ℃ for 30 s, and an extension at 72 ℃ for 30 s, with a final extension at 72 ℃ for 1 min. Finally, 321 PC samples, 87 samples from other *Pyrus* species, and two outgroup specimens were genotyped using the final panel of five mtSSR markers. Within the sampled collection and under the selected binning procedure, genotype-accumulation analysis indicated that all five loci were required to distinguish the observed multilocus mitochondrial haplotypes (Supplementary File 2; S1 Fig). The PCR products were visualized, sized, and data cleaned using QIAxcel ScreenGel software v1.2 (QIAGEN, Germantown, MD, USA). The allelic sizes were determined using a 15/600 bp alignment marker and 25–500 bp DNA size marker (QIAGEN, Hilden, Germany).

### Data binning

Raw allele-size data generated by QIAxcel were binned using the FlexiBin Excel macro [49] and used for downstream analyses. Allele sizes were assigned to allelic classes based on the repeat-motif length of each mtSSR locus. The poppr package v2.9.6 [34] in R v4.3.2 [50] was used for clone correction within populations. All subsequent population-genetic analyses used the clone-corrected dataset.

### Genetic Diversity

Genetic diversity indices were calculated across the five mtSSRs and four different populations, i.e., Asian, TNesc, SNesc, and UScult in R v4.3.2 using the package *poppr* v2.9.6. For each mtSSR locus, the number of alleles amplified (N), genetic diversity or expected heterozygosity (He), and allelic richness (Ar) were calculated using R package *hierfstat* v0.04-22 [51]. Nei’s unbiased gene diversity (H_e_) was used as the primary measure of within-population genetic variance. It was calculated across loci using the equation $He=\frac{n}{n-\text{1}}\left(\text{1}-\sum p_{i}^{\text{2}}\right)$, where *n* is the sample size and *p*_*i*_ represents the frequency of the *i*^th^ allele. Although *H*_*e*_ is conventionally termed expected heterozygosity in diploid systems, its numerical formulation for a haploid organellar marker is equivalent to haplotype diversity: the probability that two randomly sampled individuals carry different mitochondrial haplotypes. This index functions to quantify the probability that two randomly drawn individuals possess distinct maternal haplotypes, effectively removing diploid heterozygosity assumptions while remaining highly robust against homozygosity inflation artifacts. For each analyzed population, the number of individuals observed (N), the number of multi-locus genotypes (MLG) observed, the number of expected MLG (eMLG), standard error based on eMLG (SE), Shannon-Wiener Index of MLG diversity (H) [52], Simpson’s Index [53], and the number of private alleles (Pa) were calculated. Analysis of Molecular Variance (AMOVA) was performed to partition variance within and among populations using Excel add-in GenAIEx v6.5 [54].

### Population Structure using Structure and DAPC

Structure v3.4 [55] was as an exploratory Bayesian clustering approach for the genotyped PC collection. For each number of clusters (*K* = 1–5), 20 independent Markov chain Monte Carlo (MCMC) runs were performed, each with 500,000 burn-in iterations followed by 500,000 sampling iterations. Structure results were then visualized in POPHELPER structure web app v1.0.10 [56]. Evanno’s method [57] was used to identify the most strongly supported hierarchical level of KK K, and replicate runs were summarized across 20 MCMCs.

The model free multivariate clustering approach, Discriminant Analysis of Principal Components (DAPC), was also performed using the R package *adegenet* [58] to analyze the population structure of the PC dataset. The DAPC analysis was cross-validated for selecting the optimum number of principal components (PCs), with cross-validation performed using a range of 5–30 PCs based on the cumulative variance explained. Based on the lowest cross-validation error, an optimal number of 22 PCs, explaining 80% of the cumulative variance, was retained for the final analysis. The multivariate pattern was additionally visualized using an unrooted neighbor-joining tree based on pairwise Nei genetic distances [59] among the studied PC populations. FigTree v1.4.4 [60] was used to visualize and edit the phylogenetic tree.

## Results

### Characteristics of *Pyrus calleryana* mtDNA

**Fig 2:**
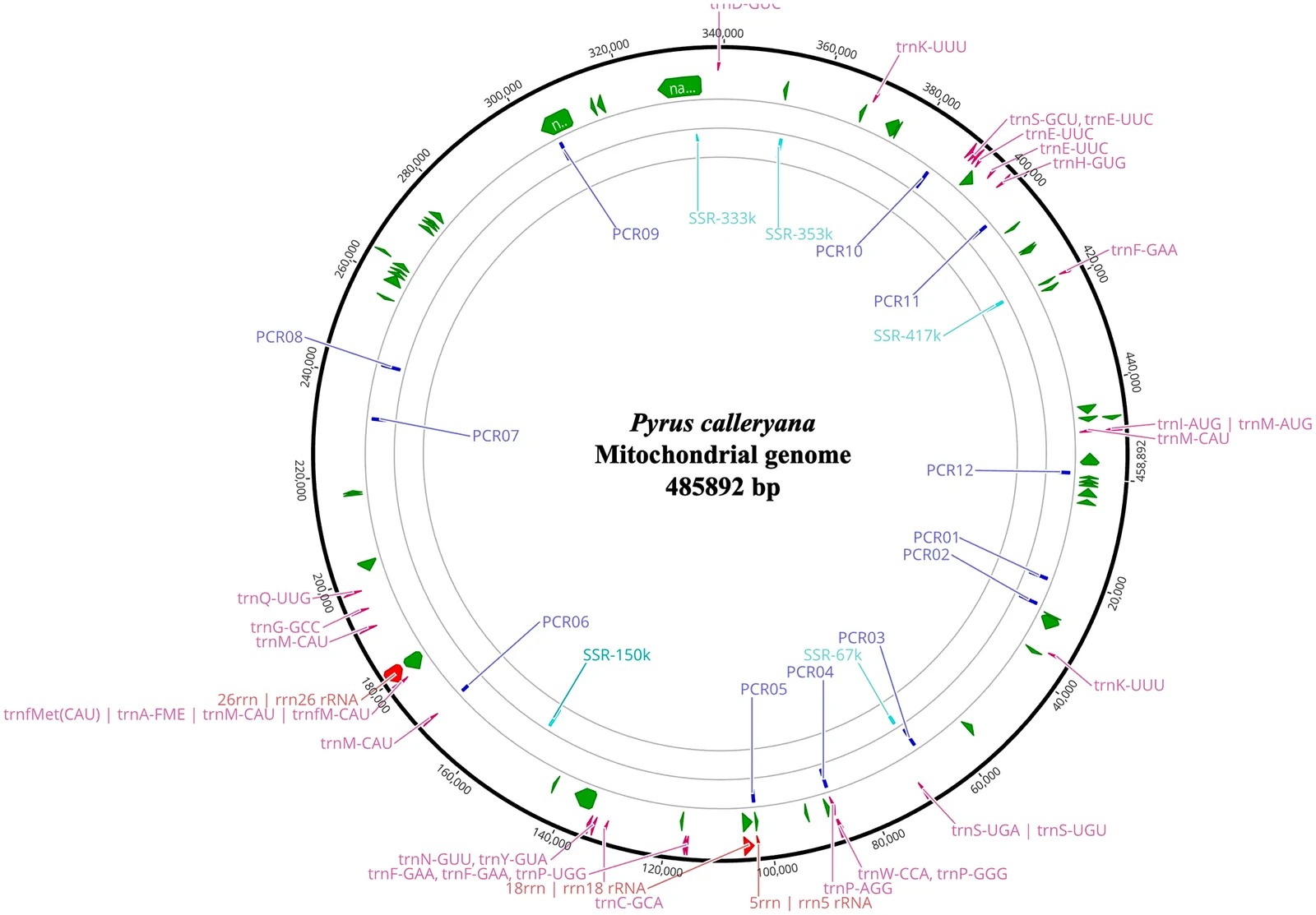
The circular map of the complete mitochondrial genome of *Pyrus calleryana.* Green arrows denote protein-coding mitochondrial genes. Pink arrows indicate t-RNAs, whereas red arrows represent r-RNAs. Blue features (PCR01…PCR12) correspond to PCR products generated by Sanger sequencing and aligned against the assembled genome for validation. Light blue labels (SSR-67k…SSR-353k) are the mtSSRs used in this study. Arrows positioned on the inner or outer tracks reflect gene orientation on the forward or reverse strand, respectively.

The selected assembly comprised a mitochondrial genome of 485,892 bp in length represented as a circular molecule with 45.21% GC content (Fig 2). NOVOwrap recovered this assembly as a single contig, and PCR and Sanger sequencing were used to support selected assembly junctions. The mitochondrial genome of PC encompassed 14 core genes, including five ATP synthase genes (*atp1, atp4, atp6, atp8, atp9*), four cytochrome C biogenesis genes (*ccmB, ccmC, ccmFc, ccmFn*), three cytochrome c oxidase genes (*cox1, cox2, cox3*), one ubiquinol cytochrome c reductase gene (*cob*), and one maturase gene (*matR*). There were three ribosomal RNAs (*rrnL, rrnS, rrn5*) detected. Among the 17 transfer RNAs (tRNAs), *trnF-GAA* and *trnM-CAU* had three copies, *trnS-UGA*, *trnK-UUU*, *trnH-GUG*, *trnG-GCC, trnC-GCA*, and *trnY-GUA* had two copies, and the remaining ones had a single copy each. The genome also had nine NADH dehydrogenase genes (*nad1, nad2, nad3, nad4, nad4L, nad5, nad6, nad7, nad9*), two large ribosome protein subunits (*rpl5, rpl10*), five small ribosome protein subunits (*rps1, rps12, rps13, rps3, rps14*), one transport membrane protein (*mttB*), and one succinate dehydrogenase gene (sdh4). The PC mtDNA showed gene content broadly similar to that reported for other *Pyrus* mitochondrial genomes, and when placed among the other genomes of the related Rosaceae species, clustered closely with other *Pyrus* species mtDNA (Fig 1).

### mtSSRs and cross-amplification

Five mtSSR loci were identified from the polished PC mtDNA assembly (Table 1). These mtSSRs comprised di- and trinucleotides with repeat numbers ranging from six to seven and total repeats length spanning 12–21 bp. All five mtSSRs amplified in the tested *Pyrus* species and in the outgroup *M. rockii* (Supplementary File 1; S2 Table).

**Table 1:** List of Short Sequence Repeats (SSRs) generated by USDA SSR finder and their respective primers generated by PrimerQuest tool.

SSR ID	Motifs	Start position in genome	End position in genome	Repeat total (bp)	Forward (F) and Reverse (R) Primers	Expected PCR product size
67k	(at)_6_	68001	68013	12	F: GTATATGTGGCACAGAGGCTAC R: TTTCATACTGTCGTATTT	200 bp
150k	(tat)_7_	150549	150570	21	F: GCAGGTATCGTATGACCCAAA R: TGCCAGTAGCTTCGTAGAATG	190 bp
333k	(tc)_6_	333964	333976	12	F: GGCCTGTAGAGCACATGTAA R: CGGCTAGGATAATTGGGAAAGA	180 bp
353k	(tc)_6_	353226	353238	12	F: GAGTAGTGAAGAATTATAGTGAGTTGTG R: GCCTTACCGGAGAGTAAGAAT	290 bp
417k	(tc)_7_	417915	417929	14	F: TTGCCTTCGCTTCCTCTTT R: TCTCTCCCATCAGGGTCATT	165 bp

### Genetic diversity

All five mtSSRs were needed to capture all the MLGs present (Supplementary file 2; S1 Fig). After clone within populations, 190 individuals out of the 321 genotyped individuals represented unique multi-locus genotypes (MLGs) or mitochondrial haplotypes. The number of individuals decreased drastically in each pre-attributed population, with the highest reduction (from *n* = 90 to *n* = 36) taking place in TNesc and the least reduction in Asian (from *n* = 72 to *n* = 65) after clone correction. The UScult group retained 41 MLGs after clone correction, indicating that some samples shared identical mtSSR profiles within their assigned population. Clone correction was therefore applied to avoid inflated representation of repeated profiles in downstream analyses. Accordingly, the clone-corrected dataset comprising190 unique MLGs was used for the remainder of the analyses. There were 2.32% missing data throughout the dataset. Locus 417k had the highest locus-level missingness (3.68%), whereas the Asian population had the highest population-level missingness (33.85%). Other populations had no missing data. The mean Shannon–Wiener index of multilocus mitochondrial haplotype diversity across the four populations was 3.83; the Asian population had the highest value (4.17), indicating high diversity of observed mitochondrial haplotypes in the clone-corrected dataset (Table 2). Simpson’s index of genotypic diversity (λ = 0.978) was high across populations, indicating a predominance of unique MLGs and relatively even genotype distribution. Overall allelic richness (A_r_) of 5.83 was observed, with UScult having the highest value of 7.49 and TNesc the lowest of 3.80. UScult also had the highest genetic diversity (H_e_ = 0.848) among the tested populations. A total of 22 private alleles were detected: 17 in the Asian population, four in UScult, one in SNesc, and none in TNesc.

**Table 2:** Genetic diversity indices observed among different *Pyrus calleryana* populations.

Population	Before CC	After CC	MLG	eMLG	% Missing	No. of Alleles	H	A_r_	λ	H_e_	E.5	P_a_
Asian	72	65	65	36	33.85	31	4.17	5.45	0.985	0.663	1	17
TNesc	90	36	36	36	0	19	3.58	3.80	0.972	0.629	1	0
SNesc	90	48	48	36	0	34	3.87	6.59	0.979	0.773	1	1
UScult	69	41	41	36	0	38	3.71	7.49	0.976	0.848	1	4
***Summary statistics***	***321***	***190***	***177***	***36***	***8.46***	***54***	***3.83***	***5.83***	***0.978***	***0.728***	***1***	***22***

Before CC: Number of individual samples before clone correction; After CC: Number of individual samples after clone correction; MLG: Multi-locus genotype; eMLG: expected multi-locus genotype; % Missing: percent of genotypes missing, i.e., those genotypes that failed to amplify; No. of Alleles: Number of alleles; H: Shannon-Wiener Index of MLG diversity [52]; A_r_: Allelic richness; λ: Simpson’s Index [53]; H_e_: Nei’s unbiased gene diversity [59]; E.5: Genotypic Evenness; Pa: Number of private alleles in each population.

An average of about 11 alleles per locus ranging from seven in locus 67k to 20 in locus 353k were detected (Table 3). The allelic richness (A_r_) across the loci ranged from 4.74 in locus 67k to 8.26 in locus 353k with an average of 5.82 which again suggests the high genetic diversity and evolutionary potential of PC populations. Nei’s unbiased gene diversity (*H_e_*; equivalent here to mitochondrial haplotype diversity) was high across the five mtSSRs (0.80–0.93; mean = 0.84), indicating substantial mitochondrial allelic diversity in the studied populations. Locus 353k exhibited the highest H_e_ (0.93), reflecting its high polymorphism and contribution to overall haplotype diversity.

**Table 3:** Genetic diversity indices of *Pyrus calleryana* based on microsatellite loci.

mtSSR Locus	No. of Alleles	% Missing	A_r_	H_e_
67k	7	1.58	4.74	0.80
150k	11	1.58	4.75	0.85
333k	10	1.58	6.34	0.83
353k	20	3.16	8.26	0.93
417k	8	3.68	5.05	0.81
***Average***	***11.2***	***2.32***	***5.82***	***0.84***

No. of Alleles: Number of alleles detected; % Missing: % of samples that failed to amplify in the given locus; A_r_: Allelic richness; H_e_: Nei’s unbiased gene diversity; [59]; for the haploid mitochondrial marker system, this metric is numerically equivalent to haplotype diversity).

### Population structure

**Fig 3:**
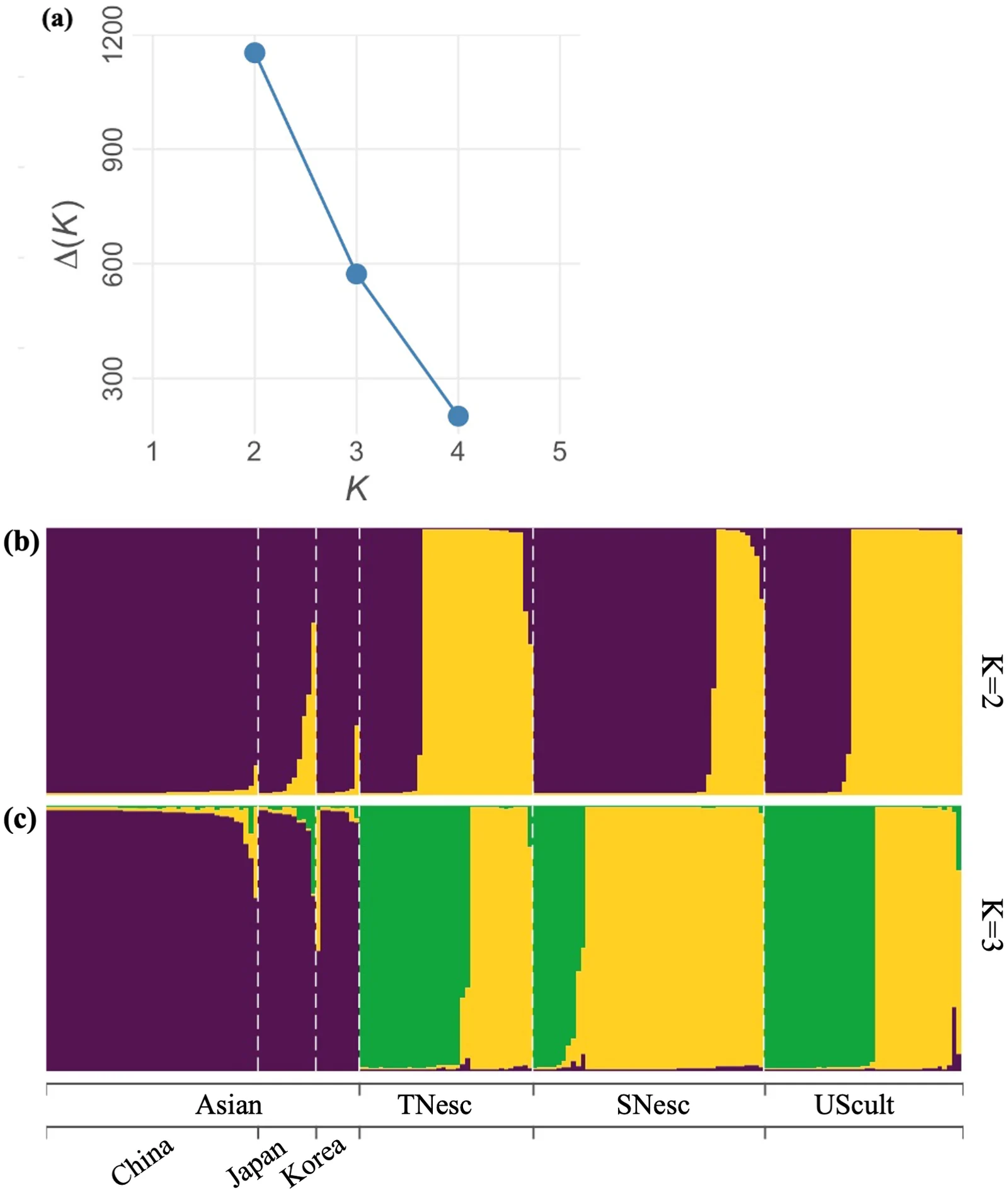
Bayesian clustering using Structure for *Pyrus calleryana* populations. Structure Bayesian clustering [55] analyzed with (a) the Evanno method [57] and visualized using (b) 2 genetic clusters (K = 2) and (c) 3 genetic clusters (K = 3). Each color indicates the estimated membership proportion of an individual in an inferred genetic cluster. ΔK is an Evanno-method statistic used to identify the strongest hierarchical level of genetic structure; it does not independently establish a unique biological number of populations. Samples are grouped by population of origin: Asian (native-range accessions from three Asian countries; China, Japan, Korea), TNesc, SNesc, and UScult. Dashed vertical lines indicate population boundaries.

Analysis of Molecular Variance (AMOVA) indicated that 17% of the observed variation occurred among populations and 83% occurred within populations, indicating that most observed variation occurred within populations, whereas 17% occurred among populations (Table 4). A ΦPT value of 0.167 (*P*<=0.001) indicated significant differentiation or population structure among the sampled populations. Exploratory Bayesian clustering using Structure indicated K=2 as the strongest hierarchical signal according to the Evanno method in the genotyped PC dataset (Fig 3a). At *K* = 2, Asian and SNesc samples showed greater membership in one inferred cluster, whereas TNesc and UScult samples showed greater membership in the other, with mixed membership profiles among several samples (Fig 3b). Additionally, when visualized at ΔK = 3, Asian remained a distinct cluster, whereas SNesc and UScult largely shared the same cluster, and TNesc was dominated by a third cluster, with UScult showing notable TNesc-SNesc admixture (Fig 3c).

**Table 4:** Analysis of Molecular Variance (AMOVA) for the *Pyrus calleryana* dataset.

Source of variation	Degree of freedom	Sum of squares	Mean squares	Est. variance	% variance	Φ_*PT*_
Among Populations	3	57.520	19.173	0.371	17%	
Within Populations	186	343.575	1.847	1.847	83%	
***Total/Average***	***189***	***401.095***		***2.219***	***100%***	0.167**

Degree of freedom (df) given by no. of samples – 1; Sum of squares: sum of squares of the deviations of all the observations from their mean; Mean squares: mean squares is the sample variance obtained by dividing the sum of squares by the respective df; Est. variance.: estimated variance; % Variance: percent of the total variance for each hierarchical level; Φ_*PT*_: partitioning of total genetic variation is the statistics calculated by the test; Significance was assessed by using 1,000 permutations of the dataset, ** = *P* < 0.001.

**Fig 4:**
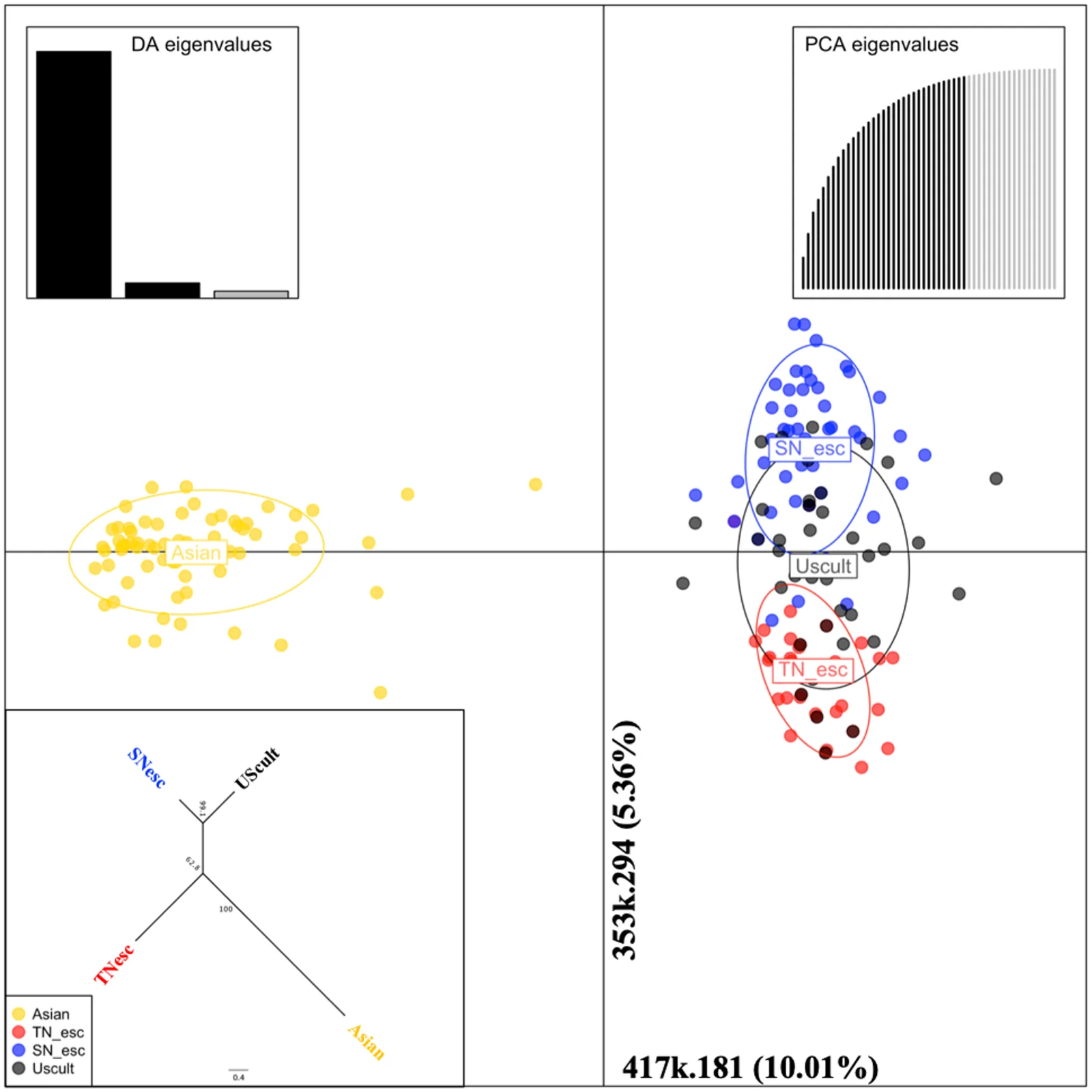
Discriminant Analysis of Principal Components (DAPC) of *Pyrus calleryana* dataset. The four populations are represented by four different colors (Orange: Asian, Red: TNesc, Blue: SNesc and Black: UScult). Twenty-two principal components, representing 80% of cumulative variance, were retained for the analysis. The alleles 353k.291 and 417k.181 contributed most strongly to separation along the displayed discriminant axes. The bottom left insert represents the genetic distance tree: Unrooted neighbor-joining tree of pairwise genetic distances (Nei) among the sampled *P. calleryana* datasets. The relative eigenvalues for the Discriminant Analysis are shown in the top‐left.

The model-free DAPC likewise separated the Asian accessions from the US-derived populations, however, the Asian population represented a complete separate cluster without admixture with the other inferred genetic cluster (Fig 4). DAPC separated the Asian accessions from the US-derived samples, which formed a second, partially overlapping assemblage. The DAPC results were also supported by the genetic distance dendrogram (Fig 4; lower left). In the DAPC and genetic-distance visualizations, UScult occupied an intermediate position between the sampled Asian accessions and the US escaped populations (TNesc and SNesc). To further resolve within-group structure, separate DAPC analyses were performed for the UScult and Asian subsets (Supplementary File 2, S2-S3 Fig). Within the UScult, samples assigned to individual cultivar names did not form discrete groups based on the five mtSSR loci, indicating that the marker panel did not uniquely resolve cultivar assignments in this collection. In the Asian-only DAPC, Japanese accessions were the most differentiated. Chinese and Korean samples showed partial separation but substantial overlap, indicating only modest within-Asia structuring.

## Discussion

After developing the novel mitochondrial genomic resource of PC, our comprehensive analyses yielded several important insights into the genetic structure and diversity of PC populations, complementing previous investigations of the same plant collection using gSSRs [11,13]. Our study documents substantial mitochondrial diversity across sampled PC populations and supports differentiation between the sampled native-range and US-derived populations.

Nuclear (gSSRs) and chloroplast simple sequence repeats (cpSSRs) have been utilized in Rosaceae species for assessing genetic diversity and phylogenetic relationships, largely due to their high transferability across species [61–63]. cpSSR primer sets developed in *Prunus* demonstrated robust cross-amplification across multiple congeners [64], thereby underscoring the utility of cpSSRs as reliable and broadly applicable markers within the family. Similarly, cpSSR resources in *Rosa* have facilitated population-level studies and varietal discrimination [65]. But, mtSSRs remain comparatively underused in Rosaceae. Previous organellar studies in *Prunus* and related genera have more commonly used mitochondrial SNPs, insertions/deletions, or CAPS markers [66]. To our knowledge, comparable cross-amplification of mtSSRs has been rarely reported in Rosaceae. Because the plastid genome of PC lacks SSRs [67], these mtSSRs provide a comparatively rapid and low-cost complement to SNP-based approaches [68–71] and for comparing genetic diversity across populations, cultivar identity, and cross-amplification potential in other *Pyrus* species. In this context, the identification of five mtSSRs from the mtDNA assembly of PC represents a novel contribution, and the mtSSRs resolved mitochondrial variation among the sampled accessions. The designed primers exhibited amplification success across different *Pyrus* species, and even into somewhat distantly related *M. rockii*, which demonstrates their taxonomically broad utility. This feature is particularly valuable for studying genetic relationships and diversity in the broader *Pyrus* genus. Furthermore, such strong cross-amplification suggests that this molecular toolbox may be useful to practitioners. Potential applications include maternal-lineage diversity assessment in *Pyrus* and related taxa, as well as preliminary lineage tracking of breeding material; definitive cultivar authentication will require additional validated markers and authenticated references as demonstrated here in the UScult for PC.

We found a high Shannon-Wiener Index of MLG diversity and Nei’s unbiased gene diversity of the PC populations we analyzed, which was similar to other works that examined the genetic diversity of PC using gSSRs [11,13,30,31]. The allelic richness observed in this dataset was within the range reported in some studies of related taxa; however, direct comparisons across studies should be interpreted cautiously because marker type, sampling design, and rarefaction procedures differ*.* For instance, in a genetic diversity study of *Malus domestica* Borkh. and *Malus sylvestris* L. using gSSRs [72,73], allelic richness was reported as ranging from 2 to 3.09, whereas in a study by Bourguiba et al., 2020 [74] in *Prunus armeniaca* L., it ranged from 8 to 13.

The elevated missing data observed in the Asian population warrants consideration when interpreting diversity estimates for this group. The Asian specimens were sourced from herbarium collections, where DNA degradation is a well-documented challenge for PCR-based genotyping [75,76]. This degradation, potentially compounded by primer binding site variation in genetically diverse native-range lineages, likely caused amplification failure at one or more loci for some individuals. Importantly, this missing data introduces a conservative bias: the true genetic diversity and private allele count of the Asian population are likely equal to or greater than the values reported here, because alleles that failed to amplify are not captured in the dataset. This missingness should be considered when comparing estimates; it may lead to underestimation of diversity and private alleles in Asian samples. The relatively high allelic richness and number of private alleles in the Asian population indicate a broad sampled base of mitochondrial diversity with unique genetic variants in the species’ native range suggesting that the native range may contain additional maternal-lineage diversity not represented in the sampled US material. A recent study of gSSRs similarly identified the highest genetic diversity in Asian populations of PC [11]. In contrast, UScult showed the highest gene-diversity estimate, which may reflect the diverse origins of material represented in the sampled cultivar collection [7,11]. Potential interspecific hybridization remains a hypothesis that requires independent verification [7,77]. Potential interspecific hybridization remains a hypothesis requiring independent verification. If interspecific hybridization has occurred, it could introduce additional maternal lineages into breeding material and thereby contribute to mitochondrial diversity. More broadly, the inter- and intraspecific hybridization and development of new cultivars are directly linked to an increase in the genetic diversity and evolution of invasiveness [6,16]. The absence of private alleles in TNesc and the single private allele in SNesc is consistent with limited numbers of population-specific maternal alleles among the sampled escaped populations. Consistent with prior gSSR studies [13], the limited number of population-specific maternal alleles among the sampled escaped populations is compatible with shared ancestry and/or historical connectivity. Because mtSSRs are maternally inherited and linked within the mitochondrial genome, they do not independently quantify nuclear gene flow, contemporary pollen-mediated exchange, or genome-wide admixture. In a self-incompatible species, such as PC, high gene flow aids in the exchange of genetic material and results in the production of viable seeds [78]. Maximum multi-locus genotypes resolution was only achieved when all five mtSSRs were used, which indicates that each marker carries essential and unique genetic information necessary for distinguishing between the genotypes. This also highlights the complexity and diversity of the PC populations.

The presence of 41 MLGs among 41 clone-corrected samples assigned to 14 cultivar names indicates that these sampled trees carried distinct mtSSR profiles (Supplementary file2; S2 Fig). The presence of these unique multi-locus genotypes could reflect distinct maternal lineages among source material, labeling or record-keeping discrepancies, or technical variation; the present data cannot distinguish among these explanations. This pattern is consistent with previously reported challenges in cultivar identity and record keeping in PC [4,11,39], but does not by itself demonstrate mislabeling. The presence of distinct mtSSR profiles among sampled cultivar-labelled trees indicates mitochondrial diversity within the UScult collection; these data alone do not establish cultivar-specific invasive potential. Potential biases introduced by the resolution of the QIAxcel capillary electrophoresis system and by allele binning, both of which inherently would collapse the detected alleles into statistically indistinguishable classes, could have affected the accuracy of allele detection to a certain extent, but the outcomes defying the expected allelic collapsing are here reported. These technical limitations should be considered when interpreting fine-scale genotype differences stemming from the multiple independently sourced and analyzed PC cultivars.

We found high within-population mitochondrial diversity in together with significant differentiation among sampled populations [11,31]. Structure identified clustering patterns that may reflect shared ancestry and/or population history. The admixture observed, especially in the SNesc and UScult populations, is consistent with shared ancestry or admixture, but organellar markers alone cannot establish ongoing hybridization or direction of gene flow. DAPC provided a complementary, model-free representation of differentiation among sampled populations, i.e., the Asian population formed a distinct cluster without admixture. This separation indicates that the sampled Asian accessions carried mitochondrial haplotypes distinct from those predominant in the sampled US-derived groups; the underlying historical processes cannot be resolved from these markers alone. One explanation is that the Asian populations represent the original genetic pool(s) [11] and the subsequent bottleneck events led to the genetic structure of US populations. Considering an alternative scenario, our sampled Asian specimens may represent only a small fraction of the PC material originally imported to the US. With extensive hybridization among multiple, now-mixed genetic sources in the US [6,11], several generations of mixing could have caused the US populations to drift away genetically from that sampled Asian subset. Consistent with the Asian sub-structuring reported with gSSRs [11], our mtSSRs recover the same pattern but with a maternal-lineage pattern broadly associated with country of origin in the sampled Asian accessions. Our findings highlight the complexity and genetic richness within PC populations. Genetic richness is essential for their conservation in at least a part of their native range where they are considered threatened [30], and for informing management strategies in the US escaped areas.

The genetic distance dendrogram and DAPC suggest the possibility of UScult occupied an intermediate position in the present DAPC and genetic-distance analyses between the Asian populations and the US escaped populations, a pattern consistent with the documented history of PC in the US [4]. This pattern may reflect the introduction history of PC in the US, where initial introductions were followed by cultivation and escapes and naturalization events, ultimately leading to distinct but related genetic clusters. However, this contrasts with previous findings of Sapkota et al., 2022 [13], where escaped populations appeared to have first diverged from the Asian population. This contrast underscores the complexity of PC’s introduction history, suggesting multiple introduction routes, differences in ancestry, and the influence of different genetic markers and sampling strategies on inferred evolutionary relationships. Notably, STRUCTURE and DAPC produced discordant placements of the SNesc population relative to the Asian group. At K = 2, STRUCTURE grouped Asian and SNesc together in one cluster, while placing TNesc and UScult in a second cluster. In contrast, DAPC separated the Asian population as a distinct cluster entirely apart from all US-derived populations, including SNesc. This discordance likely reflects fundamental differences in how the two methods handle genetic data. Because standard STRUCTURE assumptions are not fully met by linked, haploid, maternally inherited mtSSR data [55], its clustering output should be interpreted cautiously. Under these conditions, STRUCTURE may over-interpret shared ancestral alleles between Asian and SNesc as evidence of cluster membership, particularly when the number of loci is small. DAPC, by contrast, provides a complementary model-free multivariate representation of differentiation [58]; nevertheless, neither DAPC nor STRUCTURE alone resolves demographic history from a five-locus organellar marker panel. Notably, neither approach alone resolves demographic history. The separation of the sampled Asian population in DAPC is consistent with differentiation in mitochondrial lineage composition, but should not be interpreted as a definitive reconstruction of demographic history. In particular, compared with gSSRs, mtSSRs can retain maternal-lineage signals that differ from patterns captured by biparentally inherited nuclear markers and may therefore complement nuclear analyses of introduction history. Therefore, apparent shifts in the placement of UScult relative to escaped groups could reflect widespread nuclear mixing reported previously, whereas maternal haplotypes still retain signals of introduction pathways and cultivar ancestry [11,13].

In conclusion, the five mtSSR loci revealed substantial mitochondrial diversity among the sampled PC accessions. Repeated mtSSR profiles within populations while retaining 190 distinct multilocus mitochondrial haplotypes, high allelic richness, and high overall gene diversity. Both STRUCTURE and DAPC indicated differentiation between sampled Asian accessions and US-derived samples, although the placement of SNesc differed between methods. At K = 2, SNesc clustered with the Asian group, whereas TNesc and UScult formed a second cluster. Because the marker panel represents linked, maternally inherited mtDNA, these patterns should be interpreted as differences in maternal-lineage composition rather than direct evidence of contemporary admixture, pollen-mediated gene flow, or genome-wide differentiation. Cultivated and escaped US samples showed less differentiation from one another than from the sampled Asian accessions. The mitochondrial diversity observed among samples assigned to PC is consistent with distinct maternal lineages and/or cultivar-labeling or record-keeping discrepancies, which require confirmation with higher-resolution nuclear markers and authenticated reference material. For example, propagation, labeling, or record-keeping errors could result in genetically distinct plants being assigned the same cultivar name at one or more stages from production through planting. This study underscores the need for broader genomic studies to further elucidate the genetic architecture of cultivated PC composition and supports the development of robust method(s) for cultivar truthing and invasion management. Despite the valuable insights gained from mtSSRs, these results must be interpreted within the context of a single-linkage, maternally inherited organellar genome. Because our panel covers only a small fraction of the total genome, these 5 loci primarily track maternal lineage pathways and may not reflect nuclear-mediated adaptive dynamics or genome-wide selection pressures. Future research utilizing dense, nuclear high-throughput sequencing data should provide a more comprehensive assessment of genetic diversity and test for loci associated with invasive traits or selection across the broad ecological range invaded by PC. Nonetheless, the findings presented here have potential management relevance. The intermediate placement of UScult between sampled Asian and escaped populations is consistent with commercial cultivars contributing to the maternal-lineage diversity represented in naturalized populations, although these data alone do not demonstrate contemporary gene flow. These findings may inform risk assessment and management discussions in jurisdictions where Callery pear is established. They are also relevant to ongoing regulatory approaches, including sales restrictions adopted in some US states [79]. However, inferences about the effects of cultivar-specific restrictions should be tested with authenticated reference material and higher-resolution nuclear markers. Moreover, apparent discordance between cultivar labels and mtSSR profiles complicates cultivar-specific management and underscores the need for rapid molecular diagnostic tools capable of verifying cultivar identity and detecting PC in the field. The development of field-deployable assays, such as loop-mediated isothermal amplification (LAMP), which has been successfully applied for species-level identification in other systems [43,80], could provide regulatory authorities and land managers with practical species-level tools for early detection of PC and support for regulatory enforcement. Cultivar authentication, however, will require a validated multilocus nuclear-marker panel and authenticated cultivar reference material.

## Supporting information

S1 Table Metadata for *Pyrus calleryana* samples included in genetic-diversity and population-structure analyses. Includes sample identifiers, population assignments, geographic provenance, and cultivar information.
(CSV)

S2 Table Cross-amplification metadata for non-*Pyrus calleryana* samples included in genetic-diversity and population-structure analyses. Includes sample identifiers, population assignments, geographic provenance, and cultivar information.
(CSV)

S3 Table Binned, full non-clone-corrected mitochondrial microsatellite genotype dataset used in downstream population genetic analyses. Alleles are reported as binned size classes for the five mtSSR loci.
(CSV)

S4 Table PCR products used for mitochondrial-genome validation. Includes primer-pair identifiers, expected amplicon sizes, and validation information.
(CSV)

S1 Fig Genotype Accumulation Curve (GAC) for populations included within the *Pyrus calleryana* dataset. It represents the number of MLG detected (Y-axis) in relation to the number of loci (X-axis) used for genotyping. The boxes represent the interquartile ranges of the number of unique genotypes detected.
(DOCX)

S2 Fig DAPC of *P*yrus *calleryana* US cultivars. DAPC for determining the molecular variance partitioning projected using 22 Principal Components cross-checked and optimized with 1,000 permutations. Each colored dot represents US cultivar individuals.
(DOCX)

S3 FigCountry‑level STRUCTURE results for Asian *Pyrus calleryana* accessions.(A) Evanno’s ΔK statistic, with a maximum at K=2. (B) Individual ancestry coefficients for K=2, grouped by Asian country of origin (China, Japan, Korea, and Asian accessions with unknown country, NA), Tennessee escaped (TNesc), southeastern US escaped (SNesc), and US cultivar (UScult) populations. (C) Individual ancestry coefficients for K=3. Each vertical bar represents one multi-locus mitochondrial genotype, and colours indicate membership proportions in the inferred clusters. These country‑level results show the same broad clustering patterns as Fig. 3, with Asian accessions forming a largely distinct cluster and TNesc, SNesc, and UScult displaying varying degrees of admixture.(DOCX)

S4 Fig Discriminant analysis (DAPC) of *Pyrus calleryana* populations from Asian origin. DAPC for determining the molecular variance partitioning projected using 10 Principal Components cross-checked and optimized with 1,000 permutations. Each colored dot represents individuals from different countries.
(DOCX)
