# Supplementary material for: Mitochondrial genome-derived microsatellites reveal genetic diversity and population structure in Callery pear populations
Source: PLoS One. 2026 Sep 17;21(9):e0358231. doi: 10.1371/journal.pone.0358231 (PMC13585228; doi:10.1371/journal.pone.0358231)
Supplement: Supplementary file 1 [file pone.0358231.s005.docx]

**Figure S1.** **Genotype Accumulation Curve (GAC) for populations included within the *Pyrus calleryana* dataset.** It represents the number of MLG detected (Y-axis) in relation to the number of loci (X-axis) used for genotyping. The boxes represent the interquartile ranges of the number of unique genotypes detected.
