# Supplementary material for: Mitochondrial genome-derived microsatellites reveal genetic diversity and population structure in Callery pear populations
Source: PLoS One. 2026 Sep 17;21(9):e0358231. doi: 10.1371/journal.pone.0358231 (PMC13585228; doi:10.1371/journal.pone.0358231)
Supplement: Supplementary file 2 [file pone.0358231.s006.docx]

**Figure S2.** **DAPC of *P*yrus *calleryana* US cultivars.** DAPC for determining the molecular variance partitioning projected using 22 Principal Components cross-checked and optimized with 1,000 permutations. Each colored dot represents US cultivar individuals.
