# Supplementary material for: Mitochondrial genome-derived microsatellites reveal genetic diversity and population structure in Callery pear populations
Source: PLoS One. 2026 Sep 17;21(9):e0358231. doi: 10.1371/journal.pone.0358231 (PMC13585228; doi:10.1371/journal.pone.0358231)
Supplement: Supplementary file 3 [file pone.0358231.s007.docx]

**Fig. S3. Country‑level STRUCTURE results for Asian *Pyrus calleryana* accessions.** (A) Evanno’s $\Delta K$ statistic, with a maximum at $K=2$. (B) Individual ancestry coefficients for $K=2$, grouped by Asian country of origin (China, Japan, Korea, and Asian accessions with unknown country, NA), Tennessee escaped (TNesc), southeastern US escaped (SNesc), and US cultivar (UScult) populations. (C) Individual ancestry coefficients for $K=3$. Each vertical bar represents one multi-locus mitochondrial genotype, and colours indicate membership proportions in the inferred clusters. These country‑level results show the same broad clustering patterns as Fig. 3, with Asian accessions forming a largely distinct cluster and TNesc, SNesc, and UScult displaying varying degrees of admixture.
