# Supplementary material for: Mitochondrial genome-derived microsatellites reveal genetic diversity and population structure in Callery pear populations
Source: PLoS One. 2026 Sep 17;21(9):e0358231. doi: 10.1371/journal.pone.0358231 (PMC13585228; doi:10.1371/journal.pone.0358231)
Supplement: Supplementary file 4 [file pone.0358231.s008.docx]

PC1 (17.9%)

PC2 (14.8%)

**Figure S4.** **Discriminant analysis (DAPC) of *Pyrus* *calleryana* populations from Asian origin**. DAPC for determining the molecular variance partitioning projected using 10 Principal Components cross-checked and optimized with 1,000 permutations. Each colored dot represents individuals from different countries.
